# Evaluation of Cytotoxic and Anti-cancer Potential of Capsaicin on Lung Cancer Cell Line: An In Vitro Investigation

**DOI:** 10.7759/cureus.68119

**Published:** 2024-08-29

**Authors:** Shree Kathir Vel, Abinaya Ramakrishnan, Jospin Sindya, Jeevitha Rajanathadurai, Elumalai Perumal

**Affiliations:** 1 College of Medicine, Center for Global Health Research, Saveetha Medical College and Hospitals, Saveetha Institute of Medical and Technical Sciences, Chennai, IND; 2 Ophthalmology, Saveetha Medical College and Hospitals, Saveetha Institute of Medical and Technical Sciences, Chennai, IND; 3 Oncology, Center for Global Health Research, Saveetha Medical College and Hospitals, Saveetha Institute of Medical and Technical Sciences, Chennai, IND

**Keywords:** apoptosis, mtt assay, a549 cell line, anti-cancer drug discovery, human lung cancer, capsaicin

## Abstract

Background

The leading cause of cancer-related deaths worldwide is lung cancer. Approximately 1.8 million new cases were diagnosed, and 1.6 million individuals died. Available treatment options are inefficient leading to tumour recurrence. Hence there is a need for novel therapeutic advancements in lung cancer treatment. Capsaicin, a naturally occurring protoalkaloid, was found to possess several potential benefits.

Aim

The aim of the study was to examine capsaicin's cytotoxic and anti-cancer effects in the lung cancer cell line (A549).

Materials and methods

The cell viability of lung cancer cells treated with capsaicin was measured using the 3-(4,5-dimethylthiazol-2-yl)-2,5-diphenyltetrazolium bromide (MTT) assay. A549 cells were treated with capsaicin at concentrations ranging from 25 to 150 µM/mL for 24 hours. Changes in cell morphology were observed using a phase-contrast microscope. Nuclear morphological alterations in the lung cancer cells were examined through acridine orange/ethidium bromide (AO/EtBr) staining and viewed under a fluorescent microscope to identify apoptotic nuclei. Gene expression analysis was performed using quantitative real-time PCR (Polymerase Chain Reaction) to evaluate the expression of apoptotic genes, transforming growth factor-beta (TGF-β), and suppressor of mothers against decapentaplegic 2 (SMAD2). Capsaicin's anti-migratory properties were assessed using a scratch wound healing assay.

Result

Our study demonstrated that treating lung cancer cells with capsaicin dramatically decreased their vitality, with a statistically significant difference (p<0.05) between the treatment and control groups. In lung cancer cells, we measured the inhibitory concentration (IC-50) at 101.2μM/ml. Following treatment, the number of cells decreased, and those that remained exhibited cytoplasmic membrane blebbing and shrunk. With AO/EtBr staining, treated cells showed an increased number of apoptotic cells. The study's findings showed that after receiving capsaicin, there was a significant downregulation of TGF-β and SMAD2. Moreover, when compared to control cells, capsaicin-treated cells’ migration was markedly reduced. Through modification of the TGF-β/SMAD2 signaling system, capsaicin therapy dramatically promotes apoptosis and inhibits migration.

Conclusion

In conclusion, the study's results indicate that capsaicin may have anti-tumor effects on lung cancer cells. To fully comprehend the mechanism underlying capsaicin's anticancer potential and its therapeutic application, further studies are much needed.

## Introduction

Globally, over 14.1 million new instances of cancer were reported, with 8.2 million deaths [1]. The leading cause of cancer-related deaths worldwide is lung cancer. Approximately 1.6 million individuals died, and 1.8 million new diagnoses were made. Compared to women, men are more impacted. 80 - 90% of instances are thought to be brought on by smokers [2]. The increased incidence and delayed diagnosis of lung cancer in India are key concerns. The annual percentage change in the incidence of lung cancer in metropolitan regions increased significantly [3]. 70% of lung cancer patients have advanced stages of the disease when they are first diagnosed (stage III or IV) [4]. Patients with suspected lung cancer have a diagnostic examination that includes tissue diagnosis, a full staging work-up, a metastatic evaluation, and a functional patient evaluation. Sputum cytology, thoracentesis, accessible lymph node biopsy, bronchoscopy, transthoracic needle aspiration, video-assisted thoracoscopy, and thoracotomy are some of the procedures available for obtaining a histological diagnosis [5]. Treatment switches to radiation or chemotherapy when the illness reaches stages III or IV. A prospective treatment strategy to investigate in the field of lung cancer therapy is the nanomedicine delivery system, which offers an alternative to the traditional chemotherapy treatment with its numerous side effects. An innovative method of treating lung cancer is the use of nanocarrier-based medication delivery [6]. However, it is affected by the number and severity of lung tumor-specific symptoms such as coughing, discomfort, exhaustion, lack of appetite, dyspnea, and blood in the sputum. Sleep disorders affect cognitive function, but respiratory issues and weariness reduce the psychosocial component of life quality. Clinical research on the effects of chronic illnesses is gradually moving away from the idea of a purely biological assessment and toward a multifactorial assessment that takes into account a person's emotions, mood, and capacity for day-to-day functioning [7].

Therapeutic medications can have adverse effects such as toxicity, fatigue, and hair loss [8]. Plant-derived medications, known as phytochemicals, can be utilized to mitigate these issues associated with conventional treatments. Phytochemicals are non-nutritive compounds found in plants that aid in cancer treatment [9]. Some examples of plant-based anticancer medicines include paclitaxel, etoposide, vincristine, naringin, and capsaicin. These medications are made from various organic ingredients, including whole grains, fruits, vegetables, nuts, and herbs. Capsaicin is the active element in chili peppers, which are grown from plants in the Capsicum genus and are the most commonly consumed in the world. Capsaicinoids are a chemical group. Capsaicin's chemical formula is C_18_H_27_NO_3_ [10]. Capsaicin is an alkaloid, that is, odorless, colorless, hydrophobic, and extremely volatile. It has a melting point of 62-65°C and a molecular weight of 305.4 kDa [11]. This compound, also known as trans-8-methyl-N-vanillyl-6-nonenamide, is an off-white solid with crystalline, lipophilic, colorless, and odorless properties.

One of the main components of chili peppers is capsaicin. It is an effective pharmacological drug with a variety of therapeutic uses for reducing pain and inflammation, with potential benefits for treating muscular atrophy. With up to 90% absorption rates, capsaicin is absorbed passively in the gastrointestinal tract. The jejunum is the primary site of absorption, followed by the ileum and stomach. The liver is responsible for nearly all of the metabolism of capsaicin, producing several metabolites such as vanillin, vanillylamine, 5,5′-dicapsaicin, 16,17-dehydrocapsaicin, and 16- and 17-hydroxycapsaicin [11]. The vanilloid 1 receptor (TRPV1), commonly known as the capsaicin receptor, is a transient release potential (TRP) ion channel that the body uses to detect heat or temperature. Capsaicin lowers memory loss and neurodegeneration in Alzheimer's disease. There have also been reports of capsaicin's therapeutic benefits for depression and Parkinson's disease. In animal stroke models capsaicin has been shown to reduce infarction area and improve neurological outcomes [12]. Furthermore, it is found in several brain locations, including the spinal cord, astrocytes, dendrites, pericytes, and neuronal cell bodies, affecting a wide range of brain activities. Capsaicin-sensitive nociceptive neurons have capsaicin receptors in their central and peripheral branches [13].

Capsaicin has been researched extensively in various cancers, but its effects on lung cancer remain underexplored. This study aims to evaluate the anticancer potential of capsaicin on lung cancer cells in vitro.

## Materials and methods

Cell line maintenance

We obtained the normal fibroblast (3T3) and lung cancer cell lines (A549) from the National Centre for Cell Science (NCCS), Pune. Cells were grown in T25 culture flasks using Dulbecco's modified eagle medium (DMEM) supplemented with 10% fetal bovine serum (FBS) and 1% antibiotics (penicillin-streptomycin). To maintain the cells at 37°C, a humidified environment with 5% CO_2_ was used. Once 80% confluence was achieved, the cells were trypsinized and passaged.

Cell viability assay

The 3-(4,5-dimethylthiazol-2-yl)-2,5-diphenyltetrazolium bromide (MTT) test was used to assess the vitality of lung cancer cells following capsaicin therapy. The approach is based on the notion that metabolically active cells convert soluble yellow tetrazolium salt into insoluble purple formazan crystals. Plating was done in 96-well plates with 5x10^3^ A549 cells per well. Twenty-four hours after plating, cells were washed twice with 100 μL serum-free media and starved for three hours at 37°C. Following starvation, cells were treated with various concentrations of capsaicin (25-150 μM/ml). The medium from the control and capsaicin-treated cells was withdrawn after 24 hours of treatment, and 100 μL of DMEM containing MTT (0.5 mg/mL) was added to each well. For four hours, cells were maintained in a CO_2_ incubator at 37°C. After removing the MTT-containing media, the cells were washed with 1X phosphate-buffered saline (PBS, pH:7.2). Following a one-hour incubation period in the dark, 100 μL of dimethyl sulfoxide (DMSO) was used to dissolve the formazan crystals. Next, a micro-enzyme-linked immunosorbent assay (ELISA) plate reader set to 570 nm was used to measure the intensity of the color. As a proportion of control cells grown in media without serum, the number of viable cells was expressed. In the control media, there was no treatment, and cell viability was 100%. The formula for calculating cell viability is % cell viability = [A570 nm of treated cells/A570 nm of control cells]×100

Morphology analysis

For morphology analysis, 2×10^5^ cells seeded in six well plates were treated with capsaicin at the optimal dosage obtained from MTT assay (IC_50_: 101.2 µM/ml) for a whole day. The media was removed from the cells during the incubation period, and they were washed once with (pH 7.4) PBS. The plates were inspected with a phase contrast microscope.

Staining

Capsaicin's effects on lung cancer cell death were investigated using the Acridine Orange/Ethidium Bromide (AO/EtBr) dual staining approach. After a 24-hour capsaicin treatment, the cells were collected and washed in ice-cold PBS. The pellets were suspended in 1 mg/mL acridine orange and 1 mg/mL ethidium bromide (5 µL each). A fluorescent microscope was then utilized to observe the tagged cells' apoptotic changes.

Scratch wound healing test

Lung cancer cells (2×10^5 ^per well) were seeded in six well culture plates. Under an inverted microscope, a 200μl tip was used to make an incision in the cell monolayer, followed by a wash with PBS. After administering the IC_50_ dose and serum-free culture media to the treatment and control cells respectively for 24 hours, the damaged area was imaged under the same microscope. In addition, this step was repeated thrice.

Real-time PCR

The expressions of the transforming growth factor-beta (TGF-β), and suppressor of mothers against decapentaplegic 2 (SMAD2) genes were investigated by real-time PCR. The conventional technique of using Trizol Reagent (Sigma) was utilized to extract the total RNA. 2μg of RNA were reverse-transcribed to produce cDNA using the PrimeScript, First Strand cDNA Synthesis Kit (Takara Bio, Shiga, Japan). Specific primers were employed to amplify the targeted genes. The PCR was carried out using the GoTaq® qPCR Master Mix (Promega, Madison, USA), which includes SYBR green dye in addition to other PCR ingredients. A Bio-Rad CFX96 PCR equipment (Bio-Rad Laboratories, Berkeley, USA) was used to conduct RT-PCR. The comparative CT approach was employed to evaluate the data, and the Schmittgen and Livak 2−∆∆CT method was utilized to calculate the fold change.

Statistical analysis

All data obtained were analyzed by one-way ANOVA followed by Student’s t-test using PRISM software version 4 (GraphPad, San Diego, USA), represented as mean±Standard Deviation (SD) for triplicates. The level of statistical significance was set at p-value<0.05.

## Results

Pro-apoptotic potential of capsaicin on lung cancer cell line (A549) and cytotoxicity analysis of normal fibroblast cell line (3T3)

**Figure 1:**
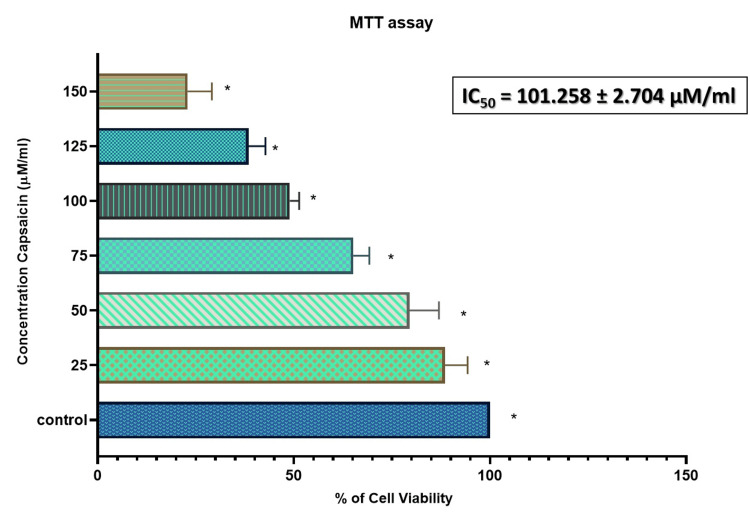
Capsaicin’s Pro-apoptotic Effect in Lung Cancer Cell Line The MTT test was used to measure cell viability after 24 hours of treatment with capsaicin (25-150μM/ml). The data is given as mean±SD (n = 3) *P-value of <0.05 indicates the statistical significance between the treatment group and the control group MTT: 3-(4,5-dimethylthiazol-2-yl)-2,5-diphenyltetrazolium bromide; IC50: 50% inhibitory concentration; SD: standard deviation

**Figure 2:**
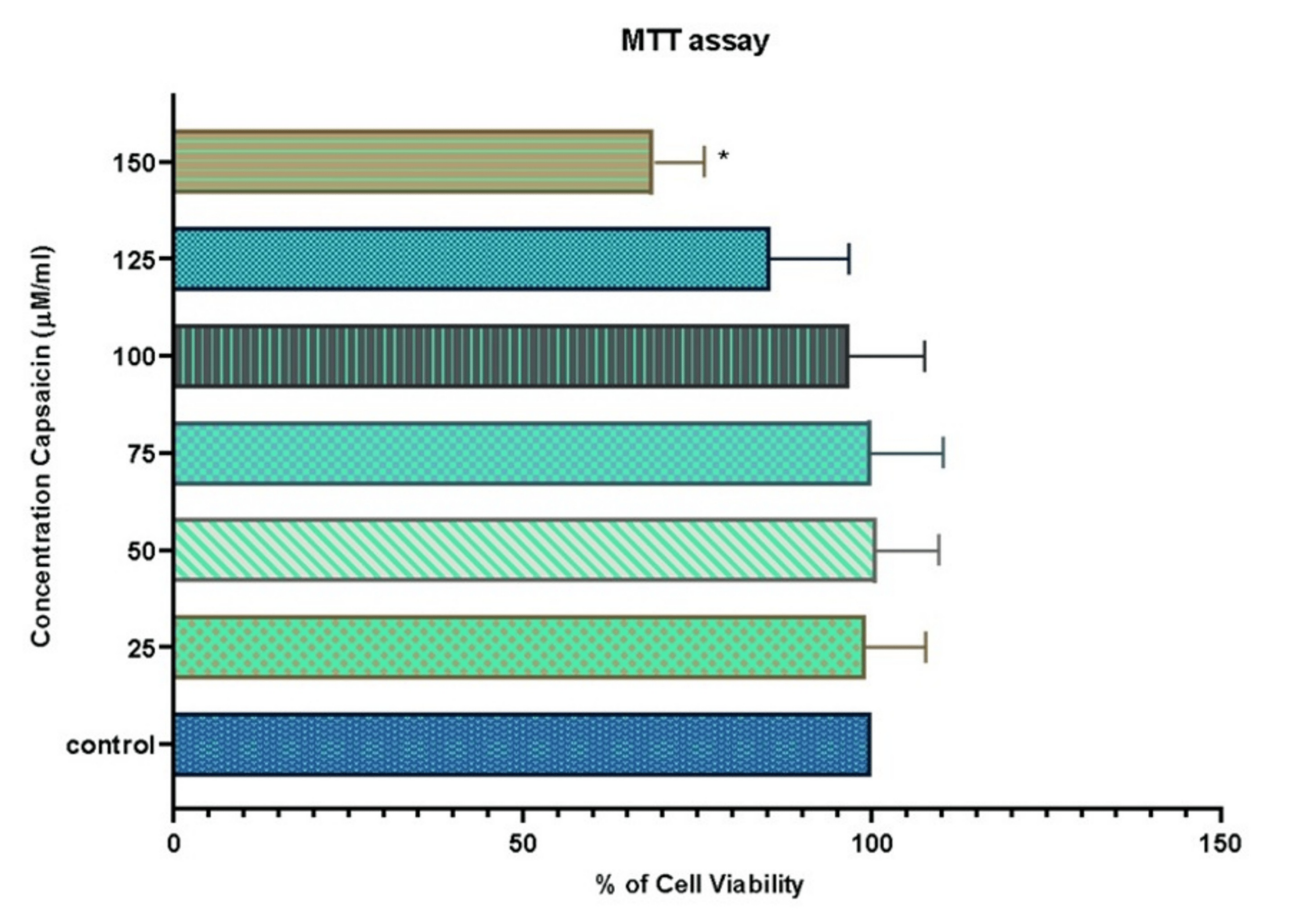
Capsaicin’s Cytotoxic Effect in Normal Fibroblast Cell Line MTT test was used to measure cell viability after 24 hours of treatment with capsaicin (25-150μM/ml). The data is given as mean±SD (n = 3) *P-value of <0.05 indicates the statistical significance between the treatment group and the control group MTT: 3-(4,5-dimethylthiazol-2-yl)-2,5-diphenyltetrazolium bromide; IC50: 50% inhibitory concentration; SD:standard deviation

For 24 hours, the cells were exposed to different doses ranging from 25 to 150 µM/ml. Our research revealed that stimulation with capsaicin extract dramatically reduced the viability of A549 Lung cancer cells at the 24-hour time point when compared to the control. As concentration increased, the quantity of viable cells steadily decreased. We saw a 50% growth inhibition at 101.2 µM/ml, which was identified as the inhibition level (IC-50) dosage value and considered for the subsequent studies (Figure 1). Normal fibroblasts, on the other hand, showed no noticeable reduction of viability after capsaicin therapy (Figure 2).

Morphology analysis

**Figure 3:**
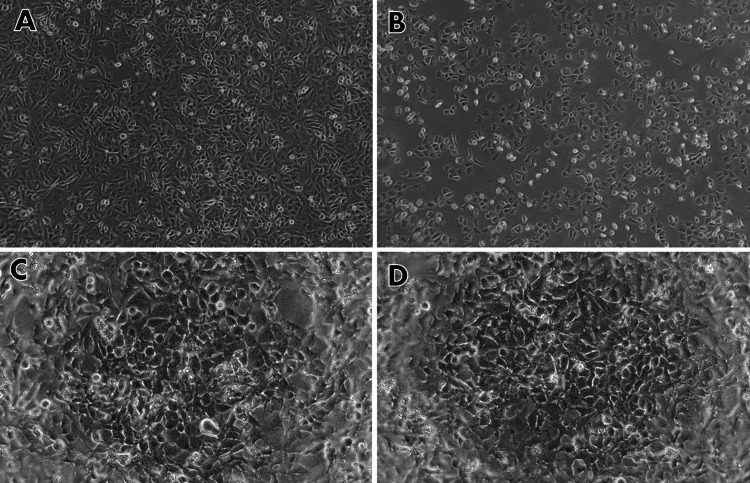
The Effect of Capsaicin on the Morphology of Human Lung Cancer Cells (A549) and Normal Fibroblast Cells (3T3) After being exposed to Capsaicin (101.2μM/mL) for 24 hours, the cells were examined using an inverted phase-contrast microscope. (A) Control cells (A549) without Capsaicin treatment. (B) A549 Cells after 24 hours of treatment with Capsaicin (101.2μM/mL) showing cell shrinkage and cytoplasmic membrane blebbing. (C) Control cells (3T3) without Capsaicin treatment. (D) 3T3 Cells after 24 hours of treatment with Capsaicin (101.2μM/mL)

After being exposed to 101.2 µM/ml of capsaicin extract for 24 hours, the A549 lung cancer cell line displayed notable morphological changes in contrast to the untreated cells. Among these changes were cell atrophy and decreased cell count, both of which are signs of apoptotic cells. Dying cells exhibited additional morphological alterations, such as rounder cells that contracted and lost contact with nearby cells. Furthermore, a few fragile cells detached from the plate surface. Nonetheless, there were no morphological changes observed in normal fibroblast cells (3T3) (Figure 3).

AO/EtBr dual staining

**Figure 4:**
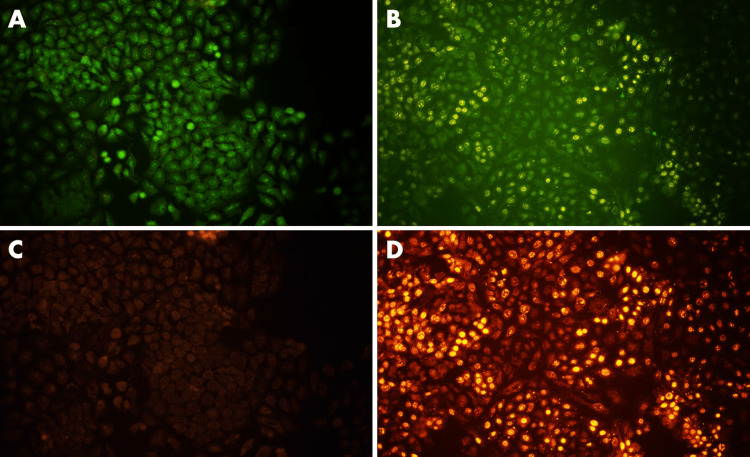
Detection of Apoptotic Cells in Capsaicin (101.2 µM/mL)-treated Lung Cancer Cells by AO/EtBr Dual Staining. Lung cancer cells were treated with capsaicin (101.2µM/ml) for 24 hours. Following treatment, the cells were subjected to dual staining with AO and EtBr. An inverted fluorescent microscope was used to obtain the images. Control cells displayed a consistent green hue and the cells treated with Capsaicin extract displayed yellow, orange, and red signals. (A) AO-stained control cells without Capsaicin treatment. (B) AO-stained cells after capsaicin treatment for 24 hours. (C) EtBr-stained control cells without Capsaicin treatment. (D) EtBr-stained cells after capsaicin treatment for 24 hours. AO: Acridine Orange; EtBr: Ethidium Bromide

After 24 hours of exposure to capsaicin (101.2µM/ml), the nuclear appearance of apoptotic cells was investigated using AO/EtBr dual staining. After 20 minutes of staining with AO/EtBr dye, the treated cells were examined under fluorescence microscopy. The observed results demonstrated that while AO tagged both living and dying cells equally showed green fluorescence, EtBr only marked cells that had lost their membrane strength. Cells dyed yellow or orange indicate that they are at apoptotic state, while cells labeled green indicate that they are still alive. In the current study, cells treated with capsaicin extract showed signals in yellow, orange, and red colors, while control cells consistently showed a green tint (Figure 4). These results show that in A549 lung cancer cells, capsaicin extract induces apoptosis.

Scratch wound healing assay

**Figure 5:**
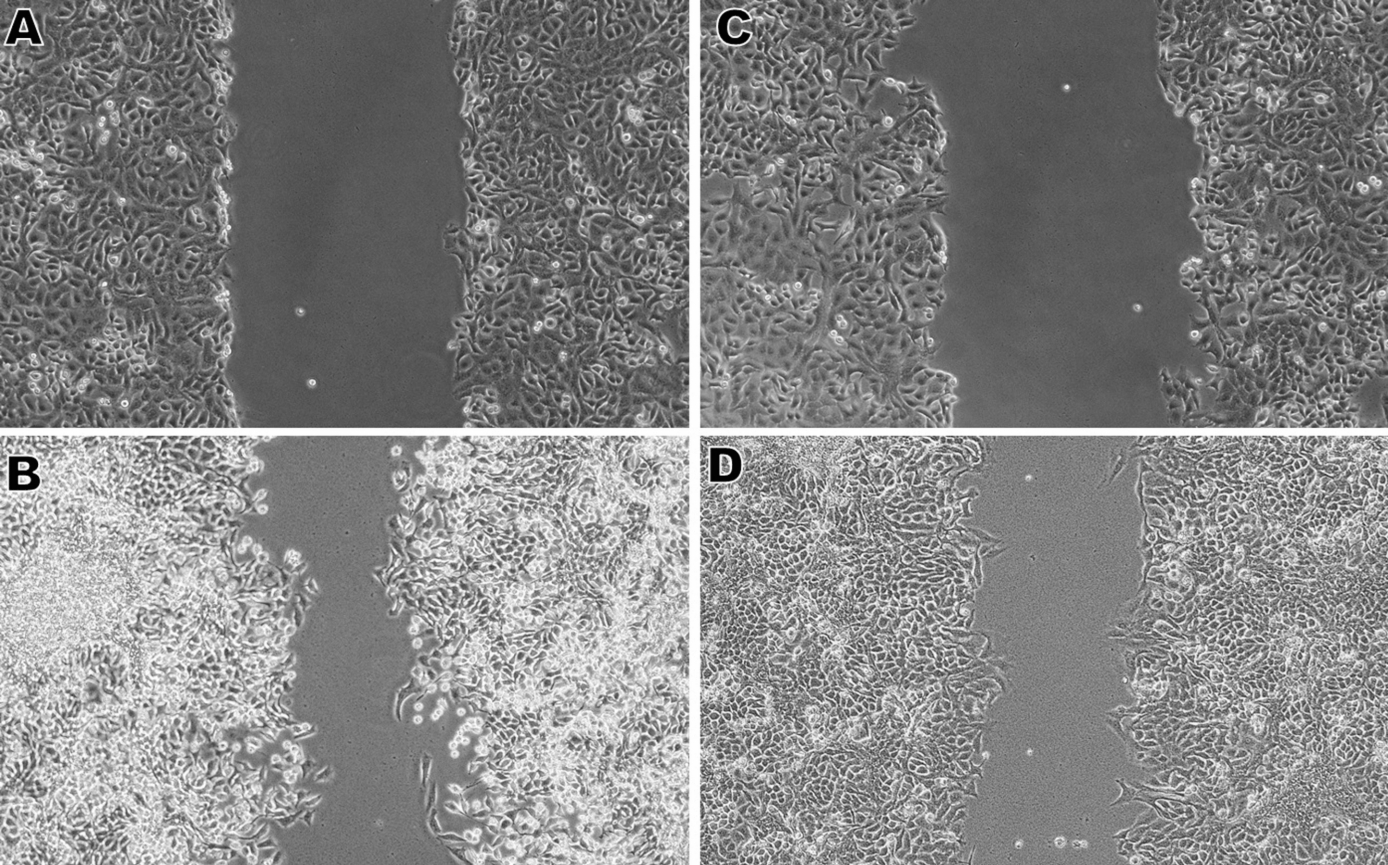
Scratch Wound Healing Assay An in vitro scratch wound healing experiment was utilized to assess capsaicin's anti-migratory properties. After damaging Lung cancer (A549) cells, a 24-hour cell migration experiment was performed with and without capsaicin (101.2µM/mL) treatment. The images were obtained using an inverted phase-contrast microscope. (A) Cells without capsaicin treatment at zero hours after scratching. (B) Deliberately migrated cells without capsaicin treatment, 24 hours after scratching. (C) Capsaicin (101.2μM/mL)-treated cells at zero hours after scratching. (D) Capsaicin (101.2μM/mL)-treated cells at 24 hours after scratch introduction showing restricted migration

Cell migration is a characteristic feature of cancer metastasis and is required for tumor invasion and dissemination. Cancer cells can migrate by altering their cytoskeletal dynamics and adhesion properties, allowing them to split from the initial tumor and invade neighboring organs. To identify potential therapeutic targets and create novel anti-cancer therapies, metastasis, and cell movement studies must be conducted in a controlled cell culture environment. A scratch test was used to determine how capsaicin affected lung cancer cell migration. The results showed that capsaicin suppresses cell migration when compared to control cells. The following observations were made. After 24 hours, untreated cancer cells in the control group had spread to cover the scratched region. Capsaicin (101.2μM/ml) significantly decreased lung cancer cell migration when compared to the control group. The cell migration distance was shorter in the capsaicin-treated group when compared to the control group (Figure 5).

Gene expression of TGFβ and SMAD2

**Figure 6:**
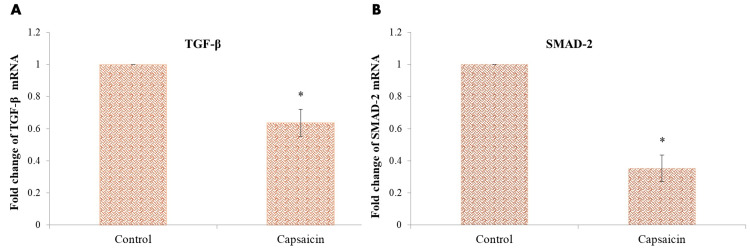
Effect of Capsaicin (101.2μM/mL) on the Gene Expression of (A) TGF-β and (B) SMAD2 in Lung Cancer Cell After normalizing target gene expression to GAPDH mRNA expression, the results are presented as a fold change from control. Each bar represents the mean±standard deviation of three different observations *P-value of <0.05 indicates statistical significance between the drug-treated and control groups TGF-β: Transforming growth factor-beta; SMAD2: Suppressor of mothers against decapentaplegic 2; GAPDH: Glyceraldehyde 3-phosphate dehydrogenase.

TGF-β/SMAD2 genes are considered the markers of A549 Lung cancer cells. Anti-apoptosis is thought to play an important role in cancer cell proliferation and metastasis. Capsaicin treatment significantly reduced the expression of TGF-β/SMAD2 anti-apoptotic genes in lung cancer cells (Figure 6). The inhibition of lung cancer cell migration was connected to the downregulation of anti-apoptotic genes. 

## Discussion

In the last several years, various research has been done to develop new chemotherapeutic drugs. Despite efforts in drug discovery, the development of novel molecules for clinical application has not increased significantly. Lung cancer has a 5-year survival rate and poor treatment outcomes despite being a very common disease. Many plant-based drugs such as naringin [14], propolis [15] noscapine [16], curcumin [17], and quercetin [18] were found to be effective in cancer treatments. Capsaicin has a variety of impacts on lung cancer cells, and this study sheds light on the potential therapeutic applications of capsaicin in the treatment of lung cancer by investigating the complicated molecular mechanisms behind these effects. These results contribute to a better understanding of capsaicin's anti-cancer effects by offering important insights into the apoptotic pathways that it influences.

This study used the 3T3 normal fibroblast cell line and the A549 lung cancer cell line to investigate capsaicin's cytotoxic and pro-apoptotic effects. The cell lines were treated with capsaicin at different dosages (25-150μM/ml) for 24 hours to assess its growth-inhibiting properties. Our data showed that capsaicin therapy significantly reduced A549 cell viability, which varied depending on the dose and duration. Capsaicin at higher concentrations (101.2 µg/ml) caused considerable cell death, suggesting possible pro-apoptotic efficacy. Phase-contrast microscopy was used to confirm the anticancer potential.

Apoptosis, also known as programmed cell death, is characterized by the activation of certain enzymes known as caspases, chromatin condensation, DNA breakage, and cell shrinkage [19]. Cancer progresses by inhibiting the apoptotic process. The most successful therapeutic technique is to induce apoptosis in tumor cells, which is used in a variety of cancer treatments. Our findings suggest a pro-apoptotic function, as Capsaicin dramatically increased the percentage of apoptotic cells. 

Capsaicin has been demonstrated in numerous studies to be helpful against a variety of cancer forms, including lung cancer [20], colorectal cancer [21], prostate cancer [22], bladder cancer [23], carcinoma of the nose [24], and cholangiocarcinoma [25]. Our findings indicate that capsaicin therapy exhibits a pro-apoptotic effect by significantly increasing the proportion of apoptotic cells. EtBr staining was employed to validate the presence of capsaicin-induced apoptotic cells in lung cancer cell lines.

Limitations

Studies on capsaicin's anticancer efficacy in vitro have limitations. They frequently fall short of accurately simulating the intricacies of the environment of the human body, including interactions with different organs and the immune system. Therefore, encouraging in vitro outcomes might not be a reliable indicator of capsaicin effectiveness and safety in living things. In order to close the knowledge gap between laboratory results and practical cancer treatment, additional studies focusing on the molecular mechanism of capsaicin's anti-cancer action as well as in vivo investigations are necessary for a more thorough knowledge of the drug's therapeutic potential.

## Conclusions

This study explored the impact of capsaicin on lung cancer cell lines, particularly focusing on the TGFβ and SMAD2 signaling pathways. The in vitro analysis demonstrated capsaicin's anticancer and anti-migratory potential, highlighting its role in modulating critical apoptotic pathways by influencing the gene expression of TGFβ and SMAD2. These findings offer valuable insights into the potential clinical applications of capsaicin in lung cancer treatment, contributing to the growing body of research on natural compounds as potential adjuvants in cancer therapy. This research underscores capsaicin's potential as a cytotoxic and pro-apoptotic phytochemical that modulates TGFβ and SMAD2 gene expression, reinforcing its potential to treat lung cancer. As various phytochemicals gain increasing recognition for their role in cancer therapeutics, further preclinical and clinical studies could establish capsaicin as a key player in lung cancer therapy.
